# AI‐Driven Dentistry and Public Health Surveillance: Opportunities and Challenges

**DOI:** 10.1002/puh2.70278

**Published:** 2026-09-10

**Authors:** Panagiotis Douris, George Kodovazenitis

**Affiliations:** ^1^ European University Cyprus School of Dentistry

**Keywords:** AI (artificial intelligence), dentistry, digital epidemiology, disparity, health surveillance, healthcare, inequalities, machine learning, public health

## Abstract

The integration of artificial intelligence (AI) into dentistry is reshaping clinical workflows, opening new possibilities for population‐level public health monitoring. Machine learning approaches, such as convolutional neural networks and other deep learning architectures, are becoming more and more capable to exhibit diagnostic performances, which are close to those of expert human readers. Beyond single‐clinic decision support, aggregated outputs that have been collected from AI diagnostic systems could be used as real‐time signals for population surveillance: When combined with geospatial and socioeconomic datasets, they may help reveal structural barriers to care. This review presents opportunities and constraints at the intersection of diagnostic AI and dental public health surveillance and outlines a five‐stage framework (data ingestion, spatiotemporal aggregation, socioeconomic enrichment, predictive modeling, and dashboard deployment) for turning de‐identified AI outputs into actionable, equity‐focused public health intelligence. This article also examines methodological, privacy, and governance challenges—including bias, interpretability, and accountability—as well as ethical issues and proposes safeguards to support equitable deployment.

## Graphical abstract

Artificial intelligence (AI) in dentistry is presented as a population‐level surveillance tool by transforming anonymized diagnostic outputs into actionable public health intelligence. A five‐stage framework integrating AI diagnostics with geospatial and socioeconomic data enables prediction of oral health disparities, supports targeted interventions, and promotes equitable resource allocation while emphasizing privacy, transparency, and ethical governance.

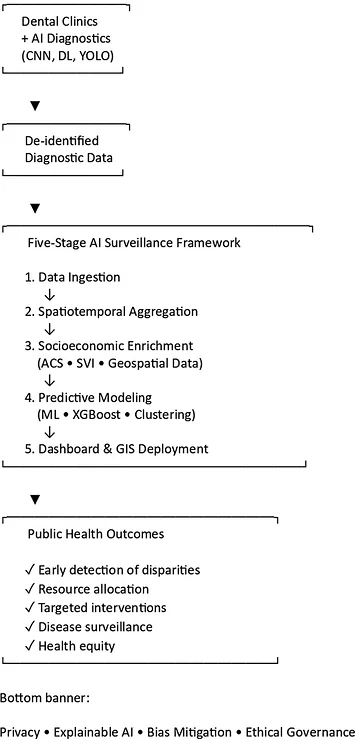

## 1 Introduction

Despite advances in diagnostic methods and treatment, large gaps in oral health persist, marked by pronounced disparities in disease prevalence, access to care, and treatment outcomes across socioeconomic and geographic groups [1, 2]. According to the World Health Organization (WHO), nearly 3.5 billion people worldwide suffer from oral diseases, many of which remain untreated due to barriers such as low income, limited education, inadequate insurance, and geographic isolation [1, 2, 3]. These challenges highlight the importance of population‐level surveillance, defined as the continuous, systematic collection and analysis of health data to monitor disease patterns, identify risk factors, and guide public health interventions.

Conventional dental public health surveillance relies on labor‐intensive screening and manual radiographic assessment, which are time‐consuming, prone to human error, and suboptimal for real‐time monitoring [4]. These limitations underscore the need for scalable, data‐driven approaches capable of capturing population‐level dynamics more efficiently [5].

Recent advancements in artificial intelligence (AI), including machine learning (ML), deep learning (DL), convolutional neural networks (CNNs), and computer vision, have brought revolutionary changes in healthcare. These technologies have demonstrated high performance in detecting caries, periodontal disease, and oral cancer while improving efficiency and standardization in clinical workflows [5, 6, 7]. Beyond clinical use, AI is increasingly being integrated into broader public health, environmental, and water monitoring systems, enabling large‐scale monitoring, sustainability assessment, and predictive analytics [8]. These interdisciplinary developments highlight the growing potential of AI‐driven approaches to facilitate real‐time, population‐level surveillance and enhance evidence‐based decision‐making in dental public health. Importantly, AI systems generate high‐throughput, standardized outputs that, when aggregated and combined with demographic, environmental, and geospatial data, could support real‐time, population‐level surveillance and more targeted public health responses [9].

Although AI is increasingly applied in public health for disease surveillance, risk prediction, and system optimization, its use in dentistry remains largely confined to individual‐level clinical decision support [5, 7]. Limited attention has been given to how AI‐generated diagnostic data can be aggregated and operationalized as population‐level surveillance signals [4]. This represents a critical gap, particularly given the influence of broader social, environmental, and structural determinants on oral health inequalities [10, 11]. Integrating AI‐driven diagnostics with public health informatics offers the potential to shift from reactive, episodic care toward proactive, data‐driven, and equity‐focused strategies. However, this transition requires careful consideration of ethical and methodological challenges, including data privacy, algorithmic bias, and equitable access [5, 7, 8, 9, 10, 11, 12, 13, 14, 15, 16].

In this context, this review aims to bridge the gap between AI‐driven dental diagnostics and public health surveillance, outlining key opportunities and challenges. As mentioned above, oral diseases are highly prevalent globally but often remain undiagnosed and untreated, highlighting the need for timely, population‐level monitoring. Although AI‐driven dental diagnostics offer high accuracy and efficiency, their full potential for public health surveillance can only be realized through a structured approach that translates de‐identified clinical outputs into actionable insights. To this end, the authors propose a five‐stage conceptual framework encompassing diagnostic data ingestion, spatiotemporal aggregation, socioeconomic enrichment, predictive modeling, and deployment through geospatial dashboards that systematically convert AI outputs into equity‐focused public health intelligence.

## 2 Novelty and Added Value

Most existing reviews of AI in dentistry focus on diagnostic accuracy and clinical decision support at the patient or clinic level [5]. This review advances the field by reframing AI‐generated dental diagnostics as a population‐level surveillance resource capable of informing public health action. Rather than treating AI outputs as isolated clinical endpoints, we conceptualize them as continuous, standardized signals that can be aggregated and analyzed to detect structural inequities in access to care. This review proposes a five‐stage operational framework that extends current AI‐based dental models beyond clinical deployment toward public health implementation. The proposed pipeline integrates dental AI with established public health informatics practices. This approach explicitly addresses translation into policy‐relevant surveillance while embedding privacy protection, bias mitigation, and interpretability within the analytical workflow. By doing so, it provides a pragmatic blueprint for leveraging dental AI to support proactive, equity‐focused public health surveillance and decision‐making.

## 3 Methods

### 3.1 Search Strategy and Data Sources

A comprehensive search was conducted across PubMed/MEDLINE, Scopus, and Google Scholar for literature published between 2015 and 2025. Using Boolean operators, we targeted keywords at the intersection of AI technology (DL, Radiomics), dentistry (Caries Detection, Periodontics, Oral Health), and public health (Epidemiology, Geospatial Analysis).

### 3.2 Inclusion and Exclusion Criteria

To maintain clinical and methodological relevance, studies were selected based on the following inclusion criteria: Peer‐reviewed research and reviews reporting AI diagnostic metrics (Accuracy, Sensitivity, Specificity) or AI‐driven population health frameworks. Exclusion criteria included non‐English publications, small‐scale case reports, and studies lacking an AI component. We extracted data regarding: (a) **Model performance**: Diagnostic metrics for dental pathology, (b) **Architectures**: Use of frameworks like U‐Net, YOLOv4, and BDU‐Net, and (c) **Public health integration**: Methods linking clinical outputs with external socioeconomic datasets (e.g., Social Vulnerability Index [SVI] and American Community Survey [ACS]). The proposed “five‐stage pipeline” was developed by synthesizing standard public health informatics workflows with dental diagnostic metadata.

## 4 Results

### 4.1 Opportunities in AI‐Driven Dentistry and Public Health Surveillance

#### 4.1.1 ML in Dental Diagnostics

AI has significantly advanced automated diagnostics in dentistry. ML models have achieved diagnostic accuracies that sometimes surpass human experts across a variety of dental conditions. For instance, CNNs have been successfully used for detecting caries with accuracies, which are higher than 90% in studies based on internal validation of radiographic datasets [17], whereas certain algorithms, such as YOLOv4 and segmentation models like U‐Net, have made possible the precise quantification of periodontal bone loss [18, 19, 20]. In a recent study, Lee et al., using external validation datasets, also demonstrated that CNNs showed strong performance in detecting caries, periodontitis, and periapical lesions radiographically [21].

**FIGURE 1:**
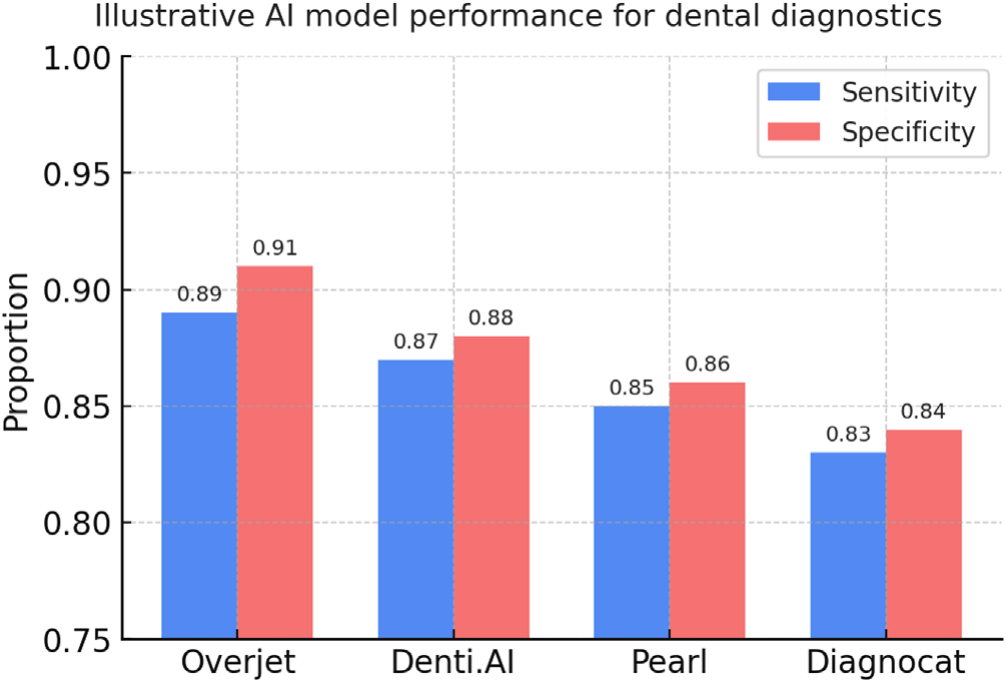
Different AI model performances in terms of dental diagnostics (illustrative). AI, artificial intelligence.

DL models like BDU‐Net showed high sensitivity and specificity in detecting various dental pathologies enabling fast disease identification which plays an important role for public health interventions on time [6, 22]. Figure 1 illustrates the performance range of commonly used AI models across dental diagnostic tasks, highlighting that most models achieve sensitivities and specificities above 0.80, with convolutional and segmentation‐based networks consistently outperforming simpler architectures. This underscores the potential for standardized, high‐throughput AI diagnostics to generate reliable data streams suitable for population‐level surveillance. Indicatively, DL models in internal and multicenter external validations have shown a specificity ranging from 0.84 to 0.91 and a sensitivity ranging from 0.83 to 0.89 (Figure 1). Liu et al. provide a comprehensive review of AI‐based dental diagnostic tools, emphasizing their potential, reproducibility, and cost‐efficiency [23]. In a recent study, Rathod et al. trained an ensemble CNN on panoramic radiographs and demonstrated diagnostic results comparable to those of human experts in detecting early‐stage pathology [24]. Yet, these powerful models remain underutilized for broader public health insight.

### 4.2 Data Analytics, Population Risk Assessment, and Public Health Prediction

When deployed across many clinics, diagnostic AI produces continuous streams of de‐identified metadata, such as counts of detected caries, periodontal bone loss measurements, diagnostic classifications (e.g., caries, periodontitis, and periapical lesions), severity gradings, timestamps, and basic geographic identifiers (e.g., postal codes or clinic locations). Aggregated across time and location, these data enable the identification of spatiotemporal clusters of elevated disease burden—such as repeated high rates of untreated caries in a postal code region—which may point to systemic access problems or service shortfalls. Aggregating ML outputs in space and time therefore creates an opportunity to treat diagnostic algorithms as epidemiological sensors: not as a substitute for clinical epidemiology, but as a complementary, high‐frequency signal that can prioritize follow‐up investigations and resource allocation at the community level.

AI applications can process and analyze large datasets of dental health and population metrics to identify patterns that might escape traditional epidemiological methods. It can thus identify trends and predict outbreaks, helping shape data‐driven public health responses, improve disease surveillance, and allocate resources appropriately [5, 17]. This function enables public health officials to plan and execute health programs and policies more effectively [7].

### 4.3 Integration With Socioeconomic and Geospatial Data—Health Promotion and Disease Prevention

The predictive power of diagnostic ML models is enhanced when integrated with demographic and socioeconomic datasets. Public repositories, such as the Global Health Observatory (GHO), the US Census Bureau, the ACS, and the SVI, offer rich metadata on insurance coverage, household income, race/ethnicity, and educational attainment—factors known to influence oral health outcomes [12].

**FIGURE 2:**
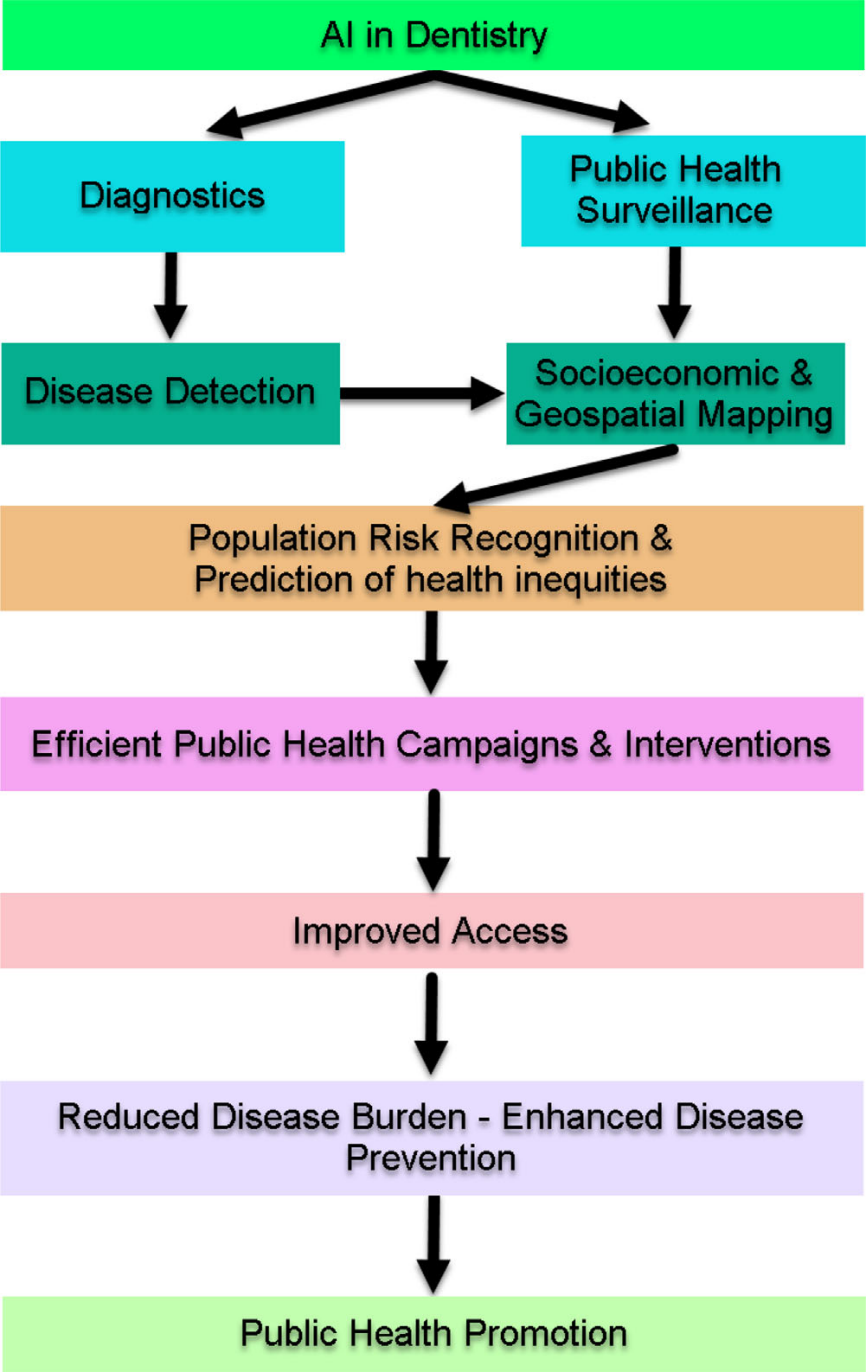
The emerging role of AI in disease prevention and public health promotion. AI, artificial intelligence.

Multimodal ML models, combining AI‐diagnosed dental data with external geodemographic indicators, offer superior performance in predicting health inequities. AI, therefore, plays a pivotal role in health promotion and disease prevention within dental public health. These systems enable personalized public health interventions, which can be directed at specific community groups based on their recognized risk profiles, enhancing the efficiency and effectiveness of public health campaigns [25, 26]. These technological tools can thus predict dental health outcomes and plan public health interventions accordingly (Figure 2).

### 4.4 Challenges and Ethical Considerations

Although the emergence of AI technology in dentistry and its impact on public health is promising, several concerns persist regarding its deployment [27, 28, 29]. Indicatively, it is mentioned that in spite of the absence of any formal consensus as far as the safe and ethical implementation of AI systems in healthcare settings is concerned, “the literature converges on several key principles of ethical AI use including transparency, justice and fairness, non‐maleficence, responsibility and privacy” [13, 15, 29]. Therefore, despite the promise, several critical challenges remain.

One major challenge is **data representation bias**: AI models trained on homogenous or proprietary datasets may embed existing biases and fail to generalize across diverse populations [14, 30, 31]. **Privacy and consent** represent another concern. Even when datasets are anonymized, large‐scale diagnostic collections can introduce privacy risks, necessitating robust safeguards and compliance with ethical and legal standards [5, 29, 32]. Techniques, such as federated learning (FL), differential privacy, and secure multiparty computation, provide mechanisms to retain analytical value while protecting sensitive data [6, 33]. Transparency in data usage and governance is critical for maintaining public trust and frameworks should address cross‐border data exchange and encryption requirements, as emphasized by the WHO [15]. Furthermore, adherence to regulations, including HIPAA in the United States, GDPR in Europe, and comparable data protection laws elsewhere, is critical to uphold trust.

**Validation requirements** also remain key: AI‐derived clinical insights must be corroborated with epidemiological ground truth to avoid false positives or misleading conclusions [6, 34]. **Interpretability** is another challenge; many AI models in healthcare often suffer from a lack of interpretability and operate as “black boxes,” because AI algorithms can be difficult to comprehend [29, 35, 36, 37]. Stakeholder adoption—especially among public health officials—requires transparent, explainable AI outputs that support actionable policy decisions [36]. Developing interpretable AI models is crucial for clinical acceptance and regulatory approval [29, 37, 38]. Finally, **cost** considerations can limit the accessibility of AI technologies in resource‐constrained settings, potentially reinforcing existing dental inequalities [5, 29, 34, 39, 40]. Thus, effective AI implementation may require significant financial investment.

To operationalize the ethical considerations discussed above, a structured mapping of core risks to corresponding mitigation strategies and governance mechanisms is presented in Table 1. This framework translates ethical principles into actionable safeguards embedded across technical, data, institutional, and policy domains. By systematically aligning each risk with defined oversight measures, the model supports the responsible and equitable integration of AI‐driven diagnostics into dental public health surveillance.

**TABLE 1:** Artificial intelligence (AI) technology and public health.

AI technology	Use case	Public health impact	Challenges	Maturity/Technology readiness
Deep CNN for radiographs	Caries detection from radiographs	Early detection → reduce treatment cost, improve access	Data diversity, generalization across populations	**Operational**: clinically validated, limited public health deployment
Natural language processing	Mining EHR clinical notes for oral health trends	Real‐time surveillance of conditions and interventions	Privacy, HIPAA/GDPR compliance	**Developing**: pilot deployments, scalability in dentistry limited
Federated learning	Multi‐institution model training without sharing patient data	Enhanced model generalizability while preserving privacy	Communication costs, model drift	**Developing**: research‐stage with limited pilots
Geospatial AI	Mapping oral disease prevalence against SVI and ACS data	Identifying underserved areas for targeted interventions	Geocoding accuracy, temporal resolution	**Developing → Operational**: operational in epidemiology, emerging in dental public health
Explainable AI (XAI)	Providing interpretable caries risk predictions	Improves clinician trust and patient understanding	Balancing interpretability with performance	**Emerging**: research‐stage, partial clinical integration

Abbreviations: ACS, American Community Survey; GDPR, general data protection regulation; HER, electronic health record; HIPAA, health insurance portability and accountability act; SVI, Social Vulnerability Index.

## 5 Conceptual Framework

For the purpose of the operationalization of AI diagnostics for the prediction of dental care disparity, a five‐stage pipeline [41] is proposed as follows:

- *Data ingestion:* Continuous capture of diagnostic outcomes from dental AI models (e.g., radiographic image classifiers).
- *Aggregation:* Spatiotemporal aggregation of diagnostic metadata, ensuring anonymization.
- *Enrichment:* Integration with public datasets (e.g., ACS, SVI, and geolocation) to contextualize clinical trends.
- *Modeling:* Use of ML models such as XGBoost, logistic regression, or spatiotemporal clustering to identify disparity zones.
- *Deployment:* Visualization through dashboards and GIS tools to guide public health responses.

**FIGURE 3:**
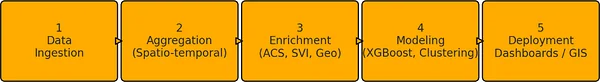
Five‐stage pipeline to operationalize AI diagnostics for dental care disparity prediction‐conceptual framework. ACS, American Community Survey; Geo, geolocation dataset; GIS, geographic information system; SVI, Social Vulnerability Index; XGBoost, an open‐source machine learning library.

Such a framework enables a shift from passive reporting to predictive public health action (Figure 3, Table 2). By systematically aggregating and contextualizing AI‐derived diagnostic outputs, health authorities can identify underserved communities, implement targeted preventive programs (e.g., mobile clinics), optimize resource allocation, and monitor equity‐related outcomes in real time. When validation procedures, privacy protections, and bias auditing mechanisms are embedded throughout each stage of the pipeline, dental AI evolves beyond a clinical decision‐support tool into a structured, equity‐oriented public health surveillance instrument.

**TABLE 2:** Five‐stage pipeline for operationalizing artificial intelligence (AI) diagnostics in dental public health.

Stage	Function	Data inputs	Practical applicability in dentistry
**1. Data ingestion**	Continuous capture of AI‐derived diagnostic outputs	Radiographic classifiers, periodontal segmentation models, and EHR metadata	Real‐time collection of caries detection rates, bone loss grading, and lesion prevalence across clinics
**2. Aggregation**	Aggregation of diagnostic metadata across time and geography	De‐identified case counts, timestamps, and postal codes	Identification of neighborhoods with persistently high untreated disease burden
**3. Enrichment**	Linkage with demographic and vulnerability indicators	ACS, SVI, insurance coverage data, and rurality indices	Contextualization of disease patterns with poverty levels, education, and service access gaps
**4. Modeling**	Detection of disparity zones and risk forecasting	Aggregated AI outputs + enriched datasets	Prediction of emerging high‐risk communities and resource allocation prioritization
**5. Deployment**	Visualization and decision‐support	Modeled outputs, risk maps, and disparity indices	Targeted mobile clinics, preventive funding programs, reimbursement reform, and outreach initiatives

Abbreviations: ACS, American Community Survey; HER, electronic health record; SVI, Social Vulnerability Index.

To date, this framework remains hypothetical. Full validation—particularly in diverse, real‐world, low‐resource, or high‐inequality settings—remains a priority for future studies. Embedding equity monitoring and post‐deployment auditing throughout these stages is critical to ensure that the pipeline functions as a coherent public health intelligence system rather than a collection of isolated technical modules.

## 6 AI Potential and Real‐World Applications

Despite the challenges, AI technology has a great and continuously evolving impact on the public health sector (Table 3). Rathod et al. demonstrated the clinical equivalence of AI diagnostic models in oral health [24]. Building upon this foundation, real‐time AI deployment in dental clinics, with anonymized outputs, can be used to inform local health departments about emergent care gaps. An example of successful AI application in dental public health is the use of deep CNNs for diagnosing periodontitis [5]. Public health agencies could further refine these approaches through partnerships with dental service organizations. This collaboration could enable the integration of AI outputs with public health programs, providing health coverage for low‐income and vulnerable population groups that claim data to triangulate service utilization, unmet need, and care accessibility.

**TABLE 3:** Ethical risks and mitigation strategies in artificial intelligence (AI)‐driven dental public health.

Ethical risk	Key mitigation measures	Governance level
Data representation bias	Diverse multi‐site datasets; inclusion of underserved populations; subgroup performance reporting; external validation	Technical and institutional
Privacy and consent	De‐identification; differential privacy; federated learning; transparent data‐use policies; regulatory compliance (e.g., GDPR/HIPAA)	Data and legal
Validation requirements	Prospective and external validation; epidemiological triangulation; continuous monitoring and model updating	Technical and institutional
Interpretability	Explainable AI methods; interpretable model architectures; transparent dashboards; human‐in‐the‐loop oversight	Technical
Cost	Public funding mechanisms; phased implementation in resource‐constrained settings; infrastructure investment	Policy and societal

Abbreviations: GDPR, general data protection regulation; HIPAA, health insurance portability and accountability act.

## 7 Discussion

AI is a promising technology that holds the potential to revolutionize the public health sector. The use of AI in dentistry encompasses a wide range of specialties such as periodontics, endodontics, orthodontics, prosthodontics, and oral surgery [42, 43, 44, 45], affecting diagnosis, treatment planning, prognosis, and patient care [25]. Additionally, AI can be useful in the production of synthetic datasets, overcoming issues present in traditionally acquired datasets. This function of AI is of particular value in the public health framework, especially in cases where there is lack of accessibility and given the fact that large datasets are diverse. Needless to mention, that certain AI algorithms, such as DL models, are known to be data intensive [26].

As healthcare technology advances, AI is expected to expand across multiple dental specialties. Radiomics exemplifies this approach, integrating genomic data, imaging, and pathology results. By combining radiographic, biological, and epidemiological data, radiomics can enhance diagnostic, prognostic, and predictive accuracy in dental care [46]. Therefore, multimodal diagnostic‐focused ML models can be harnessed to predict dental care disparities at a population scale, where anonymized diagnostic outputs can serve as proxy indicators of structural inequities.

The correct employment of AI in the healthcare sector offers the opportunity to improve access and efficiency significantly and cost‐effectively. This is of major importance, given the fact that the dental healthcare system is overwhelmed at present. AI's role in dental public health emerges as a mechanism to enhance diagnostic precision and resource allocation while simultaneously offering personalized patient interventions, positioning AI as a complementary tool rather than a complete substitute for clinical expertise [5]. AI can act as a catalyst for innovation in dental public health, enabling more proactive and individualized patient care, yet collaborative policy‐making is essential to ensure these technologies align with ethical norms and serve the societal interest.

The benefits and capabilities of AI in the healthcare sector are endless and this fact deems such a technology a powerful tool that can promote sustainable oral health. For example, AI may reduce transportation efforts and thus optimize care delivery via more self‐diagnostics and management, better triaging and referral, or supported Tele‐servicing. AI promises to increase accessibility of services for all, contributing to reducing inequalities. Nevertheless, although AI holds significant promise in enhancing workflow efficiency in dentistry, its implementation is not without challenges. One major concern involves algorithmic biases, which may arise from training datasets that do not adequately represent the diversity of the population. This fact leads to skewed results and reduced performance in underrepresented demographic groups and preserves existing inequalities [47]. Addressing these biases is crucial for ensuring equitable healthcare outcomes and maintaining trust in AI applications [48].

The presence of such biases can potentially perpetuate health disparities rather than mitigate them, especially in public health applications, where equitable access and accuracy are considered of vital importance [16]. Additionally, reliance on AI‐generated insights without thorough validation may lead to the formulation of public health policies that are misguided or not evidence‐based, potentially compromising patient care [49]. These risks highlight the need for transparent model development, ethical oversight, and continuous evaluation to ensure inclusivity and reliability in AI‐driven dental health systems [14].

Although AI integration into dental practice offers the possibility of improving early disease detection and diagnostic accuracy [50], it also carries substantial safety, reliability, and ethical concerns. Mörch et al. identified 45 ethical issues related to the use of AI in dentistry [51], such as quality of data, data ownership and privacy, data corruption, and cost of AI misdiagnosis. Given the debate surrounding what ethical AI use in dentistry entails, some authors have proposed guidelines to address this issue. For example, Duggal and Tripathi propose a five‐step “RAPID” technique. This strategy includes (R)egulation, (A)wareness, (P)olicy‐making, (I)mplementation, and (E)ducation, all with the goal of promoting and sustaining bioethical values in the use of AI in dentistry [52]. The RAPID technique forms an excellent framework for the ethical implementation of AI in dentistry and healthcare generally. This model has a strong focus on regulation of AI use, including strict guidelines for assumption of responsibility and clear protocols to maintain ethical standards [52]. Thus, the integration of AI into the public health sector and healthcare systems requires the collaboration of AI developers, healthcare professionals, and policymakers to maximize its impact.

This review has limitations. As a narrative synthesis rather than a systematic review with meta‐analysis, it may be subject to selection bias. Much of the available evidence on dental AI performance is derived from internally validated or controlled datasets, limiting conclusions about real‐world generalizability and scalability. Furthermore, the proposed surveillance framework remains conceptual and has not yet been prospectively validated in diverse, real‐life public health settings, and data on long‐term cost‐effectiveness and equity impact remain limited.

## 8 Future Directions

Future advancements in AI for dentistry must prioritize fairness, inclusivity, and clinical transparency. Developing models with diverse, high‐quality, and ethically sourced datasets is essential to minimize bias and improve generalizability across populations [31]. Moreover, robust validation mechanisms are necessary to address issues, such as misinformation and bias. Additionally, integrating explainable AI frameworks can help clinicians understand and trust the decision‐making process of algorithms, ultimately fostering safer adoption in clinical practice [37]. There is also a need for interdisciplinary collaboration among dental professionals, AI developers, ethicists, and public health experts to ensure that AI‐tools are contextually appropriate and equitable in their application [47]. Regulatory guidelines tailored to dental AI are still in their infancy and should be expanded to include continuous post‐deployment auditing, patient data protection, and accountability standards [28]. Lastly, educating practitioners and patients about AI's capabilities and limitations is critical in establishing realistic expectations and fostering responsible use in dental care.

With patient gaining access to AI tools, concerns arise regarding certification standards, digital literacy, and self‐diagnosis. Although empowering patients with information can enhance their engagement in their healthcare, it also raises concerns about misinterpretation of medical data or oversight of critical details potentially compromising patient safety. Therefore, it is important to establish guidelines and educational resources to support people in using AI as a supplementary tool rather than a substitute for professional advice.

AI in dentistry must move beyond technical refinement toward structured public health governance. Implementation should follow a phased roadmap: (i) strengthen digital infrastructure and workforce AI literacy in public clinics; (ii) pilot deployment in underserved regions; (iii) aggregate de‐identified diagnostic outputs linked to socioeconomic indicators to detect disparity hotspots; and (iv) activate targeted policy responses such as mobile services, preventive funding, and reimbursement incentives. Success should be measured not only by diagnostic accuracy but by demonstrable reductions in untreated disease and geographic inequities.

## 9 Conclusion

AI‐powered diagnostic models have achieved impressive clinical milestones in dentistry. Their potential in identifying population‐level dental care disparities represents a new frontier in preventive public health. By reframing diagnostic AI as a surveillance tool, this technology can lay the foundation for predictive, equity‐focused interventions in oral health at a population level. The integration of AI‐derived clinical data with socioeconomic and geospatial information can lay the groundwork for a proactive, efficient, data‐driven public health surveillance system in dentistry. It is via the integration of AI‐diagnosed dental indicators together with socioeconomic and spatial data that structural inequalities in oral healthcare can be detected proactively and effectively/timely tackled. As this paradigm matures, interdisciplinary collaboration between clinicians, data scientists, and public health policymakers is essential to ensure ethical, equitable, and effective deployment. Longitudinal studies are necessary to examine the long‐term impact of AI on dental public health and offer a holistic understanding of its role in advancing oral healthcare. Robust regulations and control mechanisms must be established to ensure ethical deployment for the public health's best interest.

## Author Contributions

All authors contributed to conception and design and critically revised the manuscript; all authors gave final approval and agree to be accountable for all aspects of the work. The authors have read and agreed to the published version of the manuscript.

## Funding

The authors have nothing to report.

## Ethics Statement

The authors have nothing to report.

## Consent

The authors have nothing to report.

## Conflicts of Interest

The authors declare no conflicts of interest.

## Data Availability

Data sharing not applicable to this article as no datasets were generated or analyzed during the current study.
